# Multiple waves of westward dry-land agriculture expansions along the East Silk Road during the Neolithic age

**DOI:** 10.1016/j.fmre.2025.12.013

**Published:** 2026-01-12

**Authors:** Haiming Li, Yingyu Qian, Zeli Wang, Nathaniel James, Yifu Cui, Yishi Yang, Xin Jia, Guanghui Dong

**Affiliations:** a College of Humanities & Social Development, Nanjing Agricultural University, Nanjing 210095, China; b Institution of Chinese Agricultural Civilization, Nanjing Agricultural University, Nanjing 210095, China; c Agricultural Archaeology Research Center, Nanjing Agricultural University, Nanjing 210095, China; d School of Geography, Nanjing Normal University, Nanjing 210023, China; e Institute of Environmental Archaeology, Nanjing Normal University, Nanjing 210023, China; f Department of Anthropology, University of California San Diego, San Diego 92093, USA; g College of Tourism, Huaqiao University, Quanzhou 362021, China; h Gansu Province Institute of Cultural Relics and Archaeological Research, Lanzhou 730015, China; i State Key Laboratory of Loess Science, Institute of Earth Environment, Chinese Academy of Sciences, Xi'an 710061, China; j MOE Key Laboratory of Western China’s Environmental System, College of Earth & Environmental Sciences, Lanzhou University, Lanzhou 730000, China

**Keywords:** Millet remains, The Dadiwan site, Radiocarbon dating, Dry-land agriculture expansion, Eastern Silk Road, Neolithic Age

## Abstract

The intersection between ancient agriculture, population, and language during the Neolithic is a widely researched topic across multiple disciplines. In East Asia, multiple waves of westward expansions of dry-land agriculture based on millet cultivation along the Eastern Silk Road (ESR) during the Neolithic have been identified; however, the timing and scope of these expansions remain unresolved. This is due to the uncertain timing of the earliest millet cultivation and a lack of research on the spatial-temporal patterns of this dry-land agriculture expansion. At the Dadiwan site, located in the ESR, abundant charred millet remains have been identified from associated materials in cultural layers between ∼7900 and 4800. Critically, however, direct radiocarbon dates of millet grains remain absent. Based on systematic archeobotanical analysis from the 2014–2015 excavation of the Dadiwan site, 29 samples of charred millet remains were collected and directly AMS radiocarbon dated. This yielded the earliest direct date of millet cultivation (∼7900 BP), both in the Dadiwan and across the ESR. Most of the new direct dates conflict with the previously assumed ages of the cultural strata from which the millet remains were unearthed. This highlights the importance of directly dating charred seed remains from archeological sites when exploring the chronologies of agricultural systems. We compare this new data with previously published dates, the stable isotope analysis of human diet, and archeological evidence, detecting four waves of westward expansion of dry-land agriculture along Neolithic ESR. These waves occurred approximately at 7800 BP, 6500 BP, 5500 BP and 4500 BP, broadly corresponding to cultural phases in the pre-Yangshao, early-mid Yangshao, late Yangshao-early Majiayao, and late Majiayao-Qijia periods, respectively. This work enhances our understanding of the timing and spatial patterns of dry-land agriculture, offering valuable data and insights into the connections between agricultural expansion, population growth, and language in the ancient Neolithic ESR and East Asia.

## Graphical abstract

**Figure:**
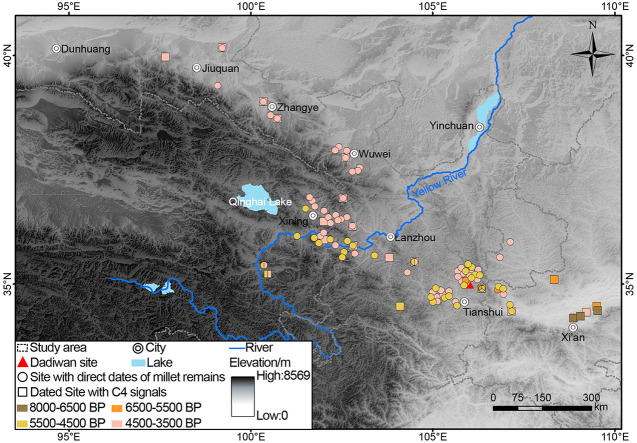

## 1 Introduction

The Neolithic origins of agriculture and its expansion across the Eurasian continent in relation to population and language diffusion are world-recognized scientific issues [[1], [2], [3], [4], [5], [6], [7], [8], [9], [10], [11], [12], [13], [14]]. The rapid development and spread of wheat and barley-based agriculture from the Fertile Crescent by 10,500 BP [[15], [16], [17]] is widely hypothesized to be a driving force in the expansion of Neolithic populations into Europe by ∼8000 BP [18,19] and South Asia by ∼7000 BP [20]. In Central Asia, genetic evidence suggests that the successful domestication of modern domestic horses contributed significantly to the rapid migration of Eurasian populations after 4200 BP [21]. At the eastern end of the Eurasian continent, the diffusion and spread of rice-based agriculture from the Yangtze River Valley in southern China after ∼10,000 BP [[22], [23], [24], [25], [26], [27]] is thought to have directly contributed to the southward expansion of Austronesian languages [[28], [29], [30], [31], [32], [33]]. In northern China, the emergence of dry-land agriculture based on the millet cultivation around 10,000 BP [6,9,[34], [35], [36]] has been posited as a crucial trigger facilitating population expansion in mid-late Neolithic [10,14,[37], [38], [39], [40], [41]]. The connections between the development of dry-land agricultural systems and the diffusion of populations and languages are subjects of intensive research [[42], [43], [44]].

The domestication of Foxtail millet (*Setaria italica*) and broomcorn millet (*Panicum miliaceum*) around 10,000 BP [6,9,[34], [35], [36]] laid the foundation for dry-land agricultural systems in China [1,[45], [46]–47]. The timing and routes of the eastward and southward diffusion of millet-based agriculture during the Neolithic have been extensively discussed [[48], [49], [50], [51], [52], [53], [54], [55]]. The two millets dispersed westward from northern China, reaching the Altai regions by 4500 BP [56,57], then the Iran Plateau by 4000 BP [58]. It remains unclear how these crops spread across the primary westward passages - the Eastern Silk Roads (ESR). The linked presence of charred millet remains and painted pottery from Neolithic sites is closely linked to dry-land agriculture; however, painted pottery is unsuitable for direct dating, making charred millet grains the ideal material for studying dry-land agriculture’s expansion and timing [59,60]. Furthermore, the stable carbon isotope analysis of human bone collagen provides an effective proxy for the consumption of C4 plants such as millets, and the same material can also be directly radiocarbon dated [61]. Therefore, integrating the direct dating of both millet grains and human bone with the stable carbon isotope analysis of human bone collagen provides a robust approach for exploring the westward expansion of Neolithic dry-land agricultural systems along the ESR.

**Fig. 1:**
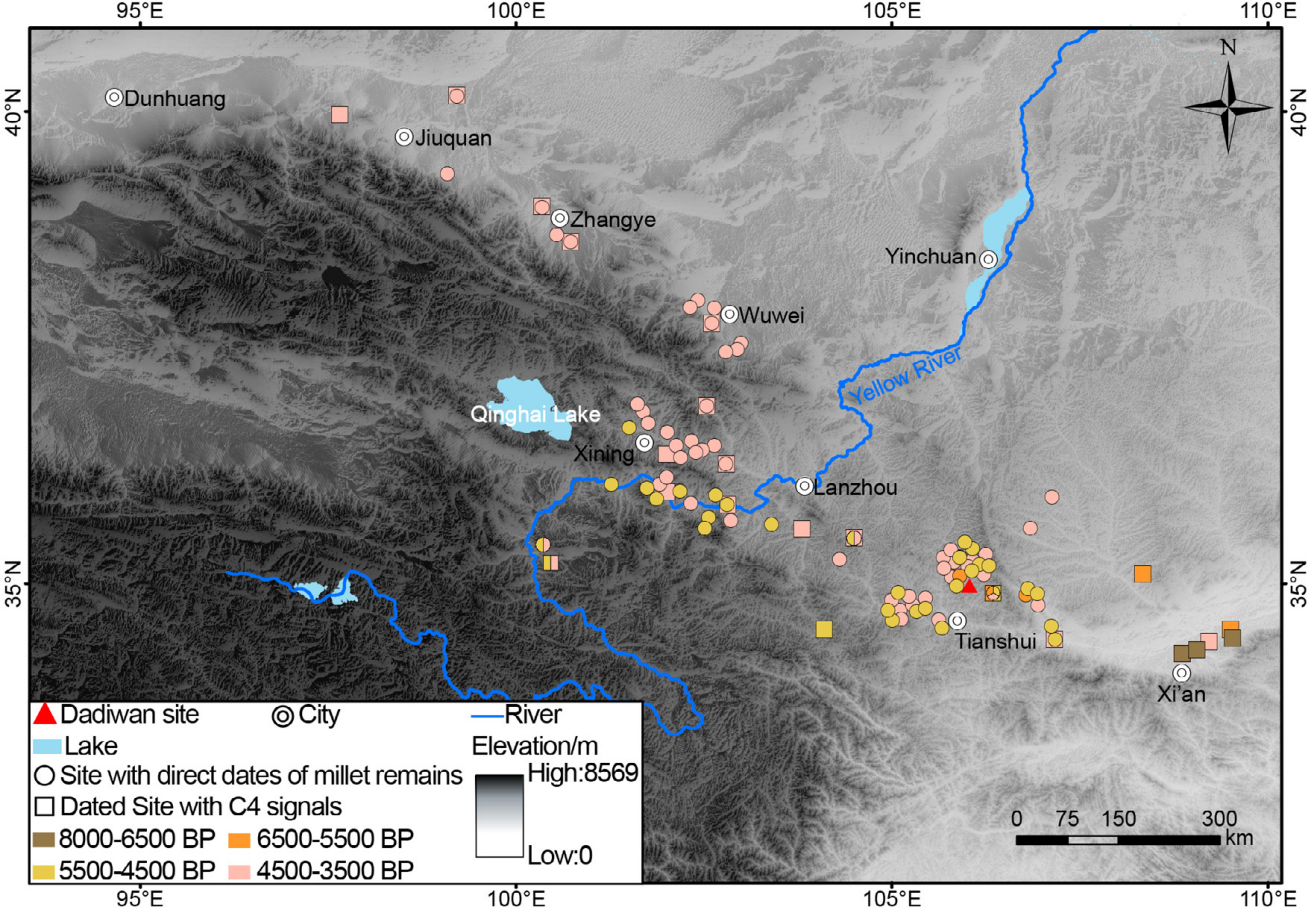
**Locations of the Dadiwan site and the sites mentioned in the text**.

Current data indicates the earliest millet remains in the ESR are found at the Dadiwan site in the western foothills of the Liupan Mountains (Fig. 1). Excavations conducted in the 1980s and 1990s, recovered eight charred broomcorn millet grains from the cultural layers of the Dadiwan I period (∼7900–7200 BP), though these grains were not directly dated [62]. Meanwhile, carbonized grains may migrate to lower and earlier cultural layers in archeological sites, “seed rain”, as evidenced by the direct radiocarbon dating of millets from archeological sites in Europe and northern China [63]. Previous archeobotanical and stable isotope evidence from Neolithic sites in the ESR has outlined the successive waves of westward expansion for dry-land agriculture [46,55,[64], [65], [66]]. Nevertheless, due to the extended occupation of the Dadiwan site between ∼7900 and 4800 BP, the potential for “seed rain” [67], and the lack of direct radiocarbon dates from archeobotanical remains, the precise timing and scope of these expansion processes remain unclear.

To address the aforementioned problem, we re-excavated the Dadiwan site in two seasons between 2014 and 2015 (Fig. S1). Macro-botanical remains were systematically collected through flotation, analyzed, and direct-AMS radiocarbon dated. We integrated this new information with published archeobotanical data, radiocarbon dates from millet remains, and the carbon isotope data of human collagen from Neolithic ESR sites (Fig. 1). The aims of this work are (1) to determine whether the radiocarbon dates of charred crop remains in the Dadiwan site correlate with the cultural stratum from which the grains were excavated; (2) to reconstruct the timing and frequency of millet utilization at the Dadiwan site to identify the earliest millet use; (3) to explore the timing and spatial scope of the westward expansion of dry-land agriculture along the ESR during the Neolithic.

## 2 Study area and site description

The Eastern Silk Roads (ESR) consist of the corridor spanning approximately 1500 km from the contemporary cities of Xi’an, Shaanxi province, to Dunhuang, Gansu province (Fig. 1). This region possesses an arid continental climate, influenced by the East Asian monsoon and the westerlies. The corridor can be divided from east to west into three primary regions: the Loess Plateau, the northeastern Tibetan Plateau, and the Hexi Corridor. The Loess Plateau region on the ESR includes the Guanzhong Basin and the Longdong and Longzhong Plateaus. Long-term alluvial erosion has shaped the Loess Plateau, creating a distinct landscape characterized by the *Yuan, Liang* and *Mao* geomorphic structures [68]. The Hexi Corridor region of the ESR extends northwest-southeast from the Wushaoling Mountains to the western border of Gansu Province, with a length of ∼1000 km and a maximum width of ∼200 km. The Hexi Corridor is primarily composed of mountains, plains, oases and plateaus. The northeastern Tibetan Plateau bordering the ESR intersects three climatic zones: the monsoonal climate of eastern China, the arid climate in northwest China, and the high cold climate of the Qinghai-Tibet Plateau [69]. The Northeastern Tibetan Plateau comprises a wide range of plateaus, mountains, valleys, deserts, and oases, in addition to containing several major drainage systems, including the upper Yellow River, Heihe, Datong, Taohe river basins, as well as the Qinghai Lake and Qaidam Basins [70]. From east to west, the overall average annual precipitation on the ESR decreases from ∼600–800 mm to ∼50–100 mm, and vegetation transitions from forest steppe to desert steppe. The average annual temperature on the ESR ranges from ∼3–14 °C, with large temperature fluctuations, and extremely low and high temperatures occurring in January and July, respectively [71,72].

The Dadiwan site (105°54′14″E, 35°0′54″N, 1460 m a. s. l.) is located near the contemporary city of Tianshui on the western Loess Plateau, with an elevation between 1458 m and 1673 m a.s.l. and a total area of about 110 × 10^4^ m^2^ (Fig. 1) [73]. Evidence indicates the site was occupied from 7800 to 4800 cal BP with five successive cultural phases: the pre-Yangshao (Phase I), Yangshao Culture (Phase II- IV) and the Lower Changshan Culture [73]. The Dadiwan site was first discovered in 1958 during a survey conducted by the Gansu Provincial Cultural Management Commission. The first major excavation of the Dadiwan site was conducted from 1978 to 1985, consisting of twelve 5 m × 5 m exploration trenches. This work uncovered successive village occupations, documenting four phases with large houses (e.g. F405, F400, H500), and finding notable artifacts from F901, a large hall-style building constructed in the fourth phase [74]. In 1995, to clarify the layout of early Yangshao village ditches, the Gansu Provincial Institute of Cultural Relics conducted a supplementary excavation of the Dadiwan site, totaling 14.95 × 10^3^ m^2^ of area [74]. This excavation uncovered 240 house sites, 325 ash pits and kiln pits, 71 tombs, 35 kiln sites, and 12 ditch sections, documenting 4147 ceramic sherds, 1931 stone tools, 2227 bone and clam tools [74]. In 2006, to investigate the transitions between Paleolithic hunter-gatherer to Neolithic agricultural economies, Lanzhou University and the Gansu Provincial Institute of Cultural Relics and Archaeology conducted a small-scale excavation in the Dadiwan site, with a single trench—Dadiwan 06. This excavation recovered 2183 pottery pieces and 877 stone tools, and obtained 11 radiocarbon and six OSL dates. These results documented a deep cultural stratigraphic sequence at Dadiwan spanning 60,000 to 5000 years ago [75].

In 2014–2015, to investigate millet cultivation and use at Dadiwan, Lanzhou University and Gansu Provincial Institute of Cultural Relics and Archeology excavated a single 6 m × 7 m unit ∼10 m in depth. The project excavated sequential 5 cm levels, documenting a total of 134 layers. The previously documented cultural stratigraphy corresponds to the 5 cm stratigraphic levels as follows: L134-L49 (Paleolithic); L48-L40 (Microlithic); L39-L33 (Dadiwan Phase I); L32-L24 (Dadiwan Phase II/early Yangshao); L23-L18 (Dadiwan Phase III/middle Yangshao); L17-L12 (Dadiwan Phase IV/late Yangshao). This recent excavation recovered a large number of animal and plant remains, providing an invaluable opportunity to study the long-term history of millet use at Dadiwan.

## 3 Materials and methods

**Fig. 2:**
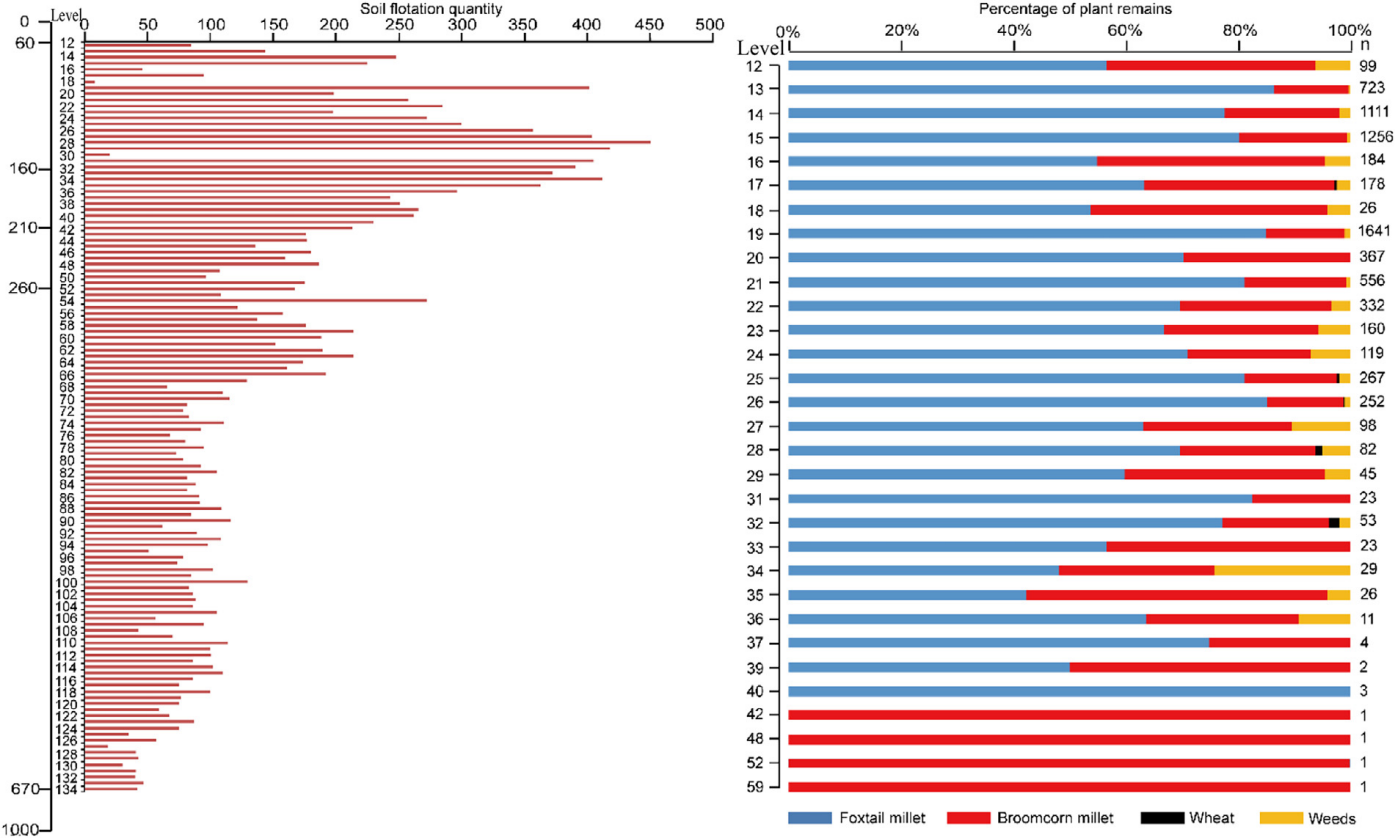
**Volume of flotation samples (left) and the percentage and count of identified plant remains (right), plotted against excavation depth at Dadiwan**.

To obtain a representative picture of Dadiwan subsistence, a systematic sampling strategy was employed during the 2014–2015 Dadiwan excavation. Neolithic strata were randomly sampled at 5 cm intervals per square meter, while Paleolithic strata were randomly sampled at 10 cm intervals per square meter. For features such as ash pits, house sites, ash ditches, storage pits, hearths, and postholes, soil samples from each were collected for flotation. A total of 1209 soil samples, totaling 18,029 liters, were collected from the 6 m × 7 m trench (Fig. 2). All samples were floated with a Flote-Tech machine-assisted flotation machine to separate the light (e.g. seeds, charcoal) and heavy (e.g. microliths, bone fragments) fractions [76]. From each sample, carbonized remains were collected with a #80 mesh (aperture size of 0.2 mm) sieve, then dried. Initial identifications of all macrobotanical remains were made with a 40 × stereo microscope at the Key Laboratory of Western China's Environmental Systems, Ministry of Education, Lanzhou University. These identifications were subsequently refined at the Paleoethnobotany Laboratory, Institute of Archeology, Chinese Academy of Social Sciences.

To establish a stratigraphic chronology for Dadiwan, twenty-nine charred millet grains and two charred wheat grains were selected for accelerator mass spectrometry (AMS) radiocarbon dating. Of these, two samples (L52 and L48) were collected from Paleolithic and microlithic cultural layers, three samples were obtained from house features, three from ash pit features, and the remaining 21 samples originated from Neolithic cultural layers (Table 1). Of the thirty-one samples, twenty-one bulk charred millet samples, two single charred wheat grains, and four single charred millet grains were measured at the Australian Nuclear Science and Technology Organization. The remaining four bulk millet samples were dated by Beta Analytic, Miami, Florida, USA. The IntCal20 curve [77] and the Libby half-life of 5568 years were used in the calculation of all dates, with calibration performed using the OxCal 4.4 program (https://c14.arch.ox.ac.uk/oxcal.html, accessed on November 20, 2024). All dates are reported as calibrated years before present (cal BP), relative to AD 1950.

**Table 1:** **Calibrated radiocarbon dates of charred crop grains from the 2014 to 2015 Dadiwan excavation**.

Lab no.	Dating method	Provenience	Data material	^14^C date (BP)	Calibrated age (cal BP)	
					1 Sigma	2 Sigma
OZS234	AMS	L12	Broomcorn millet	4565 ± 40	5251 ± 180	5241 ± 196
OZS235	AMS	L14	Foxtail millet	4620 ± 30	5376 ± 68	5379 ± 83
OZS236	AMS	L15	Broomcorn millet	4650 ± 30	5383 ± 65	5319 ± 67
OZS237	AMS	L16	Broomcorn millet	5080 ± 40	5827 ± 74	5824 ± 87
OZS238	AMS	L17	Wheat	135 ± 35	141 ± 128	141 ± 132
OZS239	AMS	L19	Foxtail millet	4585 ± 45	5260 ± 184	5249 ± 196
OZS 240	AMS	L20	Foxtail millet	4495 ± 35	5167 ± 115	5143 ± 158
OZS 241	AMS	L22	Foxtail millet	5040 ± 40	5810 ± 80	5782 ± 119
Beta420473	AMS	F1 Floor4	Broomcorn millet	4930 ± 30	5655 ± 51	5659 ± 59
OZS 243	AMS	L25	Foxtail millet	4495 ± 35	5167 ± 115	5143 ± 158
OZS 244	AMS	L26	Foxtail millet	4625 ± 30	5378 ± 68	5380 ± 81
OZS 245	AMS	L26	Foxtail millet	4700 ± 30	5447 ± 121	5449 ± 128
OZS 246	AMS	L26	Wheat	250 ± 30	232 ± 75	288 ± 137
OZS 247	AMS	L27	Foxtail millet	4535 ± 35	5187 ± 120	5181 ± 130
Beta420474	AMS	F2	Broomcorn millet	5570 ± 30	6354 ± 41	6353 ± 52
OZS 248	AMS	L28	Foxtail millet	4415 ± 35	4964 ± 81	5068 ± 202
OZT293	AMS	F2	Broomcorn millet	5005 ± 30	5756 ± 94	5770 ± 117
OZS 249	AMS	L29	Broomcorn millet	4480 ± 30	5162 ± 116	5134 ± 155
OZS 250	AMS	L31	Foxtail millet	4610 ± 35	5373 ± 72	5275 ± 189
OZS 251	AMS	L31	Foxtail millet	4265 ± 35	4843 ± 14	4801 ± 146
OZS 252	AMS	L32	Foxtail millet	4380 ± 35	4948 ± 76	4950 ± 90
OZS 253	AMS	L32	Foxtail millet	4410 ± 35	4963 ± 78	5065 ± 201
OZS 255	AMS	L34	Foxtail millet	4475 ± 45	5161 ± 121	5134 ± 166
OZS 256	AMS	L35	Broomcorn millet	4310 ± 35	4896 ± 58	4899 ± 65
OZS 257	AMS	L35	Foxtail millet	4425 ± 35	5049 ± 162	5073 ± 202
Beta420476	AMS	H7	Foxtail millet	4430 ± 30	5074 ± 181	5075 ± 200
OZT 294	AMS	L38	Broomcorn millet	4485 ± 35	5163 ± 116	5137 ± 157
Beta420475	AMS	H5	Foxtail millet	4450 ± 30	5121 ± 147	5089 ± 196
OZT291	AMS	H7	Foxtail millet	4760 ± 40	5526 ± 56	5459 ± 130
OZT295	AMS	L48	Broomcorn millet	6930 ± 60	7757 ± 68	7794 ± 133
OZT297	AMS	L52	Foxtail millet	4695 ± 40	5446 ± 121	5448 ± 130

^±^Refers to standard deviation.

## 4 Results

**Fig. 3:**
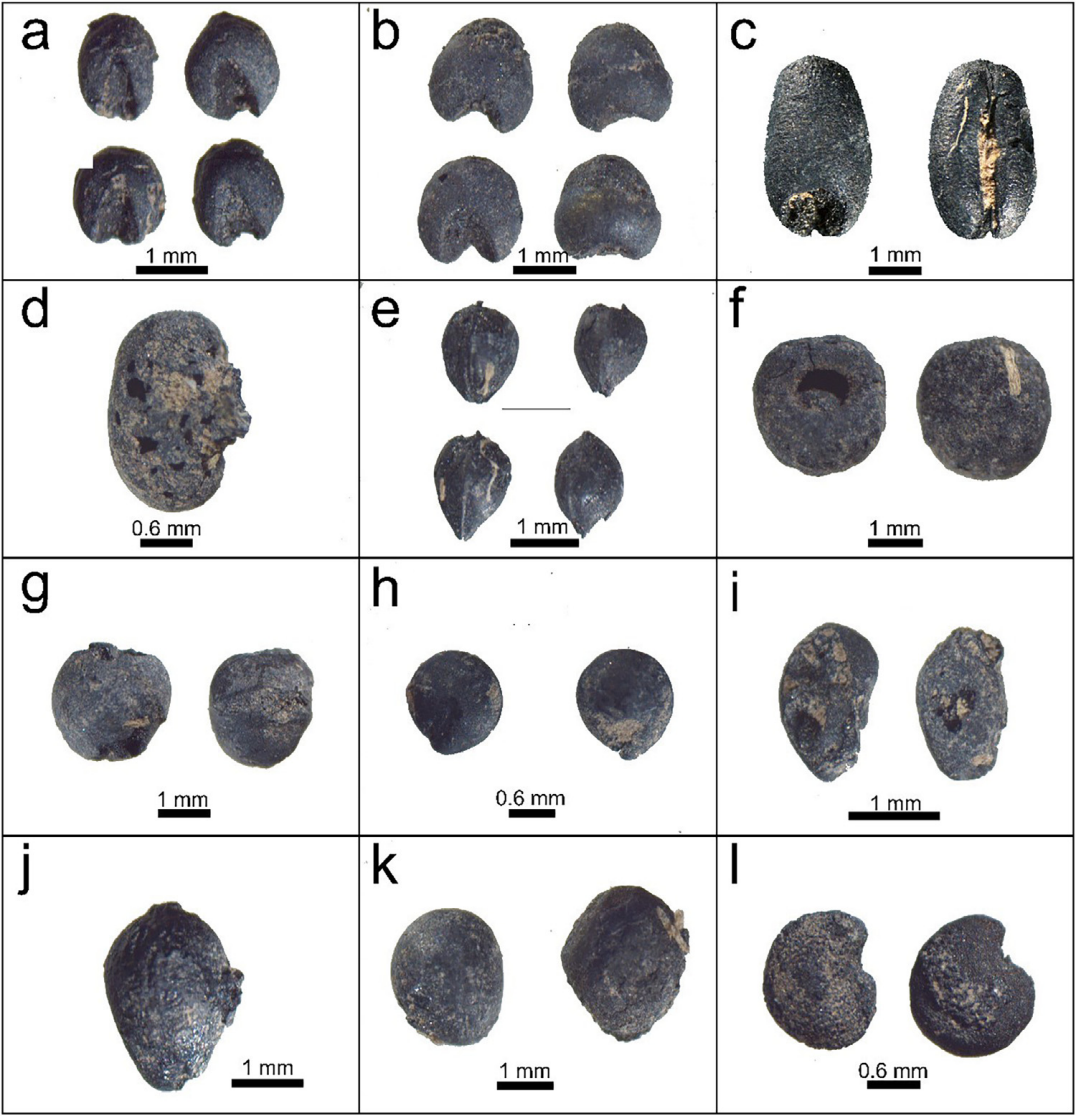
**Photographs of identified plant remains from Dadiwan.** (a) foxtail millet (*Setaria italica*); (b) broomcorn millet (*Panicum miliaceum*); (c) Wheat (*Triticum aestivum*); (d) Wild Soybean (*Glycine soja* Siebold & Zucc.); (e) Garden Sorrel (*Rumex acetosa* L.); (f) Threehorned Badstraw (*Galium tricorne* Stokes); (g) Broadleaf Vetch (*Vicia cracca* L.); (h) Lambsquarters (*Chenopodium album* L.); (i) Daghestan Sweetclover (*Melilotus suaveolens* Ledeb.); (j) Sea-buckthorn (*Hippophae rhamnoides*); (k) Ural Licorice (*Glycyrrhiza uralensis* Fisch.); (l) Erect Milkvetch (*Astragalus adsurgens* Pall.).

A total of 25,424 charred plant seeds were identified from all 1209 soil samples during the excavation of the Dadiwan site in 2014–2015 (Table S1; Fig. 2). Those plant remains were assigned to 17 plant taxa consisting of three crops: foxtail millet (*Setaria italica*) (Fig. 3a), broomcorn millet (*Panicum miliaceum*) (Fig. 3b), and wheat (*Triticum eastivum*) (Fig. 3c). One foxtail millet and three broomcorn millet grains were identified from the Paleolithic and Microlithic cultural layers (L59-L42) (Table S1). In Neolithic strata, foxtail millet and broomcorn millet seeds account for 58.7% and 38.2% of total plant remains (Table S1). In addition, 1115 weed or wild plant seeds representing 14 plant taxa were also identified, accounting for 4.39% of the total assemblage. Identified taxa include Wild Soybean (*Glycine soja* Siebold & Zucc.) (Fig. 3d), Green Bristlegrass (*Setaria viridis* (L.) Beauv.), Ereck Mlikvetch (*Astragalus adsurgens* Pall.) (Fig. 3l), Daghestan Sweetclover (*Melilotus suaveolens* Ledeb.) (Fig. 3i), Lambsquarters (*Chenopodium album* L.) (Fig. 3h), Nepal Knotweed (*Polygonum nepalense* Meisn.), Garden Sorrel (*Rumex acetosa* L.) (Fig. 3e), Ural Licorice (*Glycyrrhiza uralensis* Fisch.) (Fig. 3k), and Threehorned Badstraw (*Galium tricorne* Stokes) (Fig. 3f). The absolute counts of all carbonized seeds from all samples are listed in Table S1 and Fig. 2, with images presented in Fig. 3.

Radiocarbon dates of all 29 charred millet samples collected from the Paleolithic-Neolithic layers are shown in Table 1. These 29 radiocarbon dates suggest that the Dadiwan site was occupied between 7800 and 4800 cal BP (2 sigma). Of the 29 directly dated millet samples, only one charred broomcorn millet grain from the microlithic layer (L48) dates to the pre-Yangshao period (7922–7691 cal BP). A single carbonized broomcorn millet sample from the house features (F2) dates to the early Yangshao period (6405–6301 cal BP). The remaining 27 millet radiocarbon dates range from 6000 to 4800 cal BP (Table 1). Notably, 10 of the AMS-dated millet samples are inconsistent with the relative dates of their associated cultural layers (Fig. 3). For example, a foxtail millet sample from an early microlithic layer (L52) instead corresponds to the late Yangshao period (5578–5318 cal BP). Similarly, a foxtail millet sample from Dadiwan Ⅰ layer (L38), dates to the late Yangshao period (5294–4980 cal BP) (Table 1; Fig. 3). Further, two wheat grains excavated from Neolithic layers L17 and L26 are clearly intrusive, yielding recent dates (141 ± 132 and 288 ± 137 cal BP), corresponding to the Ming and Qing Dynasties (AD 1368–1911).

## 5 Discussion

### 5.1 The chronology and frequency of millet utilization at the Dadiwan site during the Neolithic age

**Fig. 4:**
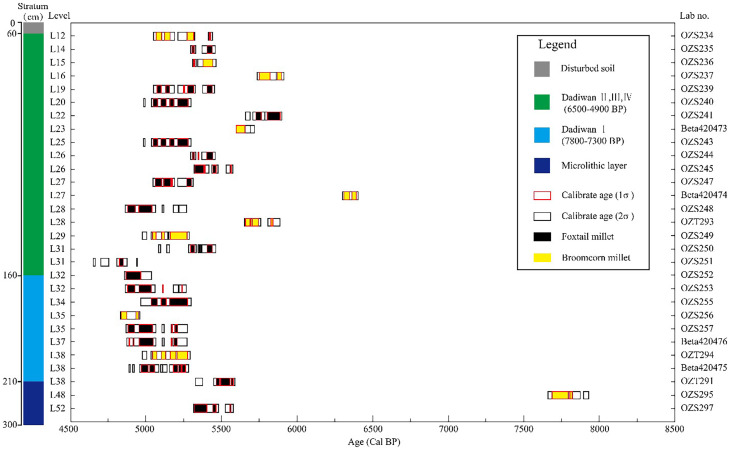
**Comparison between the direct dates of millet remains and the relative cultural strata containing those samples at the Dadiwan site**.

In the past 2 decades, advancements in the precision of accelerator mass spectrometry (AMS) ^14^C dating, combined with the refinement of systematic archeobotanical research methods, have enriched our understanding of the timing of crop domestication and spread of agricultural systems [[10], [11], [12],^37^,^40^,^41^,^47^,^57^,^61^,^63^,^65^,[78], [79], [80], [81], [82], [83], [84], [85], [86]]. Systematic archeobotanical analysis and the direct dating of 29 millet samples indicate that the human occupation and millet use between 7900 and 4800 cal BP (2 sigma). This analysis suggests broomcorn millet use began approximately 7900–7200 cal BP (Tables 1; Fig. 4). A single carbonized broomcorn millet grain from microlithic layer (L48) was directly dated to between 7922 and 7691 cal BP (Table 1; Fig. 4), indicating that the earliest human use of broomcorn millet at this site dates to ∼7900 cal BP. In addition, only 41 carbonized broomcorn millet grains were unearthed from nearly 12,427 liters of flotation soil sampled from Paleolithic and Dadiwan I cultural layers (L134–33) (Fig. 2; Table S1). This low ubiquity and density, in addition to the previous evidence of only eight carbonized broomcorn millet grains recovered from the Dadiwan I strata (7900–7200 BP) [62], suggests the limited use of broomcorn millet by site inhabitants during the pre-Yangshao period. Furthermore, the stable isotope analysis of human bone collagen samples from the Baijia site (∼7500–6500 cal BP), in the Wei River Basin of the Loess Plateau, found a mixed C3/C4 diet, supporting only the occasional use of C4 plants during the pre-Yangshao period [87]. This consumption of C4 plants was likely millet, although this conclusion cannot be certain as plant remains were not recovered from the site [87]. Archeobotanical evidence from other sites also supports this interpretation. Direct dating of millet from three sites in northern China—Zhuzhai at Zhengzhou in Henan (7639 ± 46 cal BP) [83], Xinglonggou at Chifeng in Inner Mongolia (7642 ± 29 cal BP) [9], and Yuezhuang at Changqing in Shandong (7745 ± 78 cal BP) [80]—confirms the presence of millet between 7823 and 7593 cal BP. This evidence collectively suggests that ancient peoples in northern China began occasionally utilizing millet and developing dry-land agricultural subsistence strategies by ∼7900 cal BP.

However, it should be noted that some of the charred grains recovered from Dadiwan were intrusions into earlier stratigraphic levels. For instance, two wheat grains retrieved from cultural layers (L26 and L17), associated with cultural strata dating between 7900 and 4800 cal BP, yielded radiocarbon dates that were significantly more recent, corresponding to the Ming and Qing Dynasties (AD 1368–1911) (Table 1; Fig. 4). This discrepancy is similar to the Jiahu site in Wuyang, Henan Province, where three carbonized wheat seeds recovered from early cultural layers (9000–8200 BP), yielded dramatically different radiocarbon dates revealing that the grains ages were no earlier than 800 cal BP [88]. The late occurrence of wheat at Dadiwan is consistent with studies indicating that southwest Asian crops, such as wheat and barley, were not introduced to China until around 5200 cal BP [57].

This issue is also present in the millet samples at the Dadiwan site. AMS dating of 10 out of 29 millet samples found radiocarbon dates that conflict with the dates of relative cultural strata (Fig. 4). In one instance, a charred foxtail millet seed sample from Microlithic layer (L52) dates to the late Yangshao period (5578–5318 cal BP). Similarly, a sample from the Dadiwan I layer (L38, 7900–7200 BP), produced an AMS date firmly dating to late Yangshao (5294–4980 cal BP) (Table 1; Fig. 4). This pattern is likely caused by the vertical migration of grains between stratigraphic levels, driven by bioturbation and geophysical processes like soil cracking [89], a phenomenon that is common at archeological sites [61,62,78,81,84,90]. Nevertheless, despite this “seed rain”, consistent radiocarbon dates provide robust evidence that broomcorn millet was domesticated and utilized by humans before 7900 cal BP. These further foregrounds the danger of relying on indirect dates for archeobotanical grains, even when samples are from reliably provenanced cultural layers.

This analysis of archeobotanical and AMS ^14^C data suggests a period of occasional use of broomcorn millet (c. 7900–6500 BP), followed by the intensified cultivation of both foxtail and broomcorn millet (c. 6500–4800 cal BP). This interpretation is supported by the large numbers of carbonized foxtail millet (*n* = 5960) and broomcorn millet (*n* = 1502) that have been recovered from the Dadiwan Phases II, III, and IV cultural layers (Table S1; Fig. 2). Direct radiocarbon dates further refine this chronology. The majority of charred millet grains (28 out of 29) from the Dadiwan site yielded AMS results all younger than 6500 cal BP. A single carbonized broomcorn millet grain from the house sites feature (F2) was directly dated to 6405 and 6299 cal BP; however, the remainder (27 out of 29) ranged between ∼6000 and 4800 cal BP (Table 1; Fig. 4). These findings indicate that the frequent use of millet at Dadiwan likely did not occur earlier than 6500 cal BP.

This chronology is consistent with evidence from nearby archeological sites. Archeobotanical evidence from the Gedachuan site similarly indicates that the intensive human utilization of foxtail and broomcorn millet did not begin before 6000 cal BP [91]. In addition, stable isotope evidence of human diet from Gedachuan found a gradual increase in C4 plant consumption, suggesting that significant millet utilization did not start until 6300 cal BP [52]. At Dadiwan, stable isotope evidence from human and animal bone collagen also indicates an increasing reliance on C4 plants, likely millet, after 5900 cal BP [67]. Moreover, 27 out of the 29 AMS dating data from Dadiwan site are concentrated after 5900 cal BP, despite the noted inconsistencies between stratigraphic layer and radiocarbon date (Table 1; Fig. 4). From this integrated archeobotanical and isotopic data, we argue that the frequent use and cultivation of millet by Dadiwan peoples began between 6500 and 5500 cal BP, increasing significantly between 5900 and 5500 cal BP.

The large-scale use of millets at Dadiwan, particularly the large-scale use of the higher-yielding foxtail millet, likely began at c. 5500 cal BP. A total of 3334 carbonized millet grains, accounting for 44.13% of all millet remains across all contexts were unearthed from the cultural layers of Dadiwan Phase IV (L17-L12) (Table S1; Fig. 2). Simultaneously, despite the inverted relationship between age and stratum, 22 out of 29 directly dated millet samples from the Neolithic strata (L40-L12) at Dadiwan date to between 5500 and 4800 cal BP. This concentration of dates indicates the increasing use of millet grains during this time (Fig. 4; Table S1). Moreover, 18 of these samples are foxtail millet, although this high proportion may reflect the selection of samples for dating (Fig. 4; Table S1). The results from Dadiwan are consistent with published data from the nearby Gedachuan site, which indicate increasing foxtail millet consumption after 5500 cal BP [52,91]. At Gedachuan, the proportion of foxtail millet in site assemblages exceeded that of broomcorn millet after ∼5500 BP [91]. Human and faunal stable carbon isotope data from Gedachuan also suggest that humans and animals were heavily dependent on C4 millet crops in the western Loess Plateau between ∼5500 and 4300 cal BP [52]. This regional trend is also supported by evidence at Dadiwan, where phytolith and starch evidence from pig dental calculus, and the stable isotope analysis of millet grains demonstrate the presence of an intensive millet–livestock system by at least 5500 cal BP [92]. Similar trends have been observed at other Western Loess Plateau sites, including Zhuanglang [93], Gangu [94], and Dingxi [37]. This cumulative evidence leads us to argue that the large-scale utilization of millet, particularly on the higher-yielding foxtail millet, occurred between 5500 and 4800 BP at Dadiwan.

In short, our archeobotanical analysis and direct radiocarbon dating of millet from the Dadiwan site suggest that the earliest use of broomcorn millet by ancient humans likely dates to ∼7900 cal BP, though with limited use between 7900 and 7200 cal BP. After 6500 cal BP, millet use gradually became more frequent, until between 5500 and 4800 cal BP the higher-yielding foxtail millet came to dominate human and animal subsistence in the region.

### 5.2 Multiple waves of westward dry-land agriculture expansions along the Eastern Silk Road during the Neolithic age

The cultural packages originating in North China have had a profound impact on social development as they expanded and spread westward [46,54,55,[64], [65], [66]]. These “packages” are identified by the presence of millet, human skeletons exhibiting C4 dietary signatures, and painted pottery [54]. Therefore, by integrating these three lines of evidence, we can gain a more comprehensive understanding of the spread of dry-land millet agriculture [11,46,54,67,[95], [96], [97]]. Dadiwan, as a key archeological site that spans the origin and intensification of dry-land millet agriculture across the ESR, enables a comprehensive reconstruction of this agricultural system—from emergence to intensification—through the systematic archeobotanical studies and direct dating of millet remains. By integrating archeobotanical, isotopic, and material cultural evidence, this study seeks to track the westward expansion of dryland agriculture following the Neolithic intensification of millet.

**Fig. 5:**
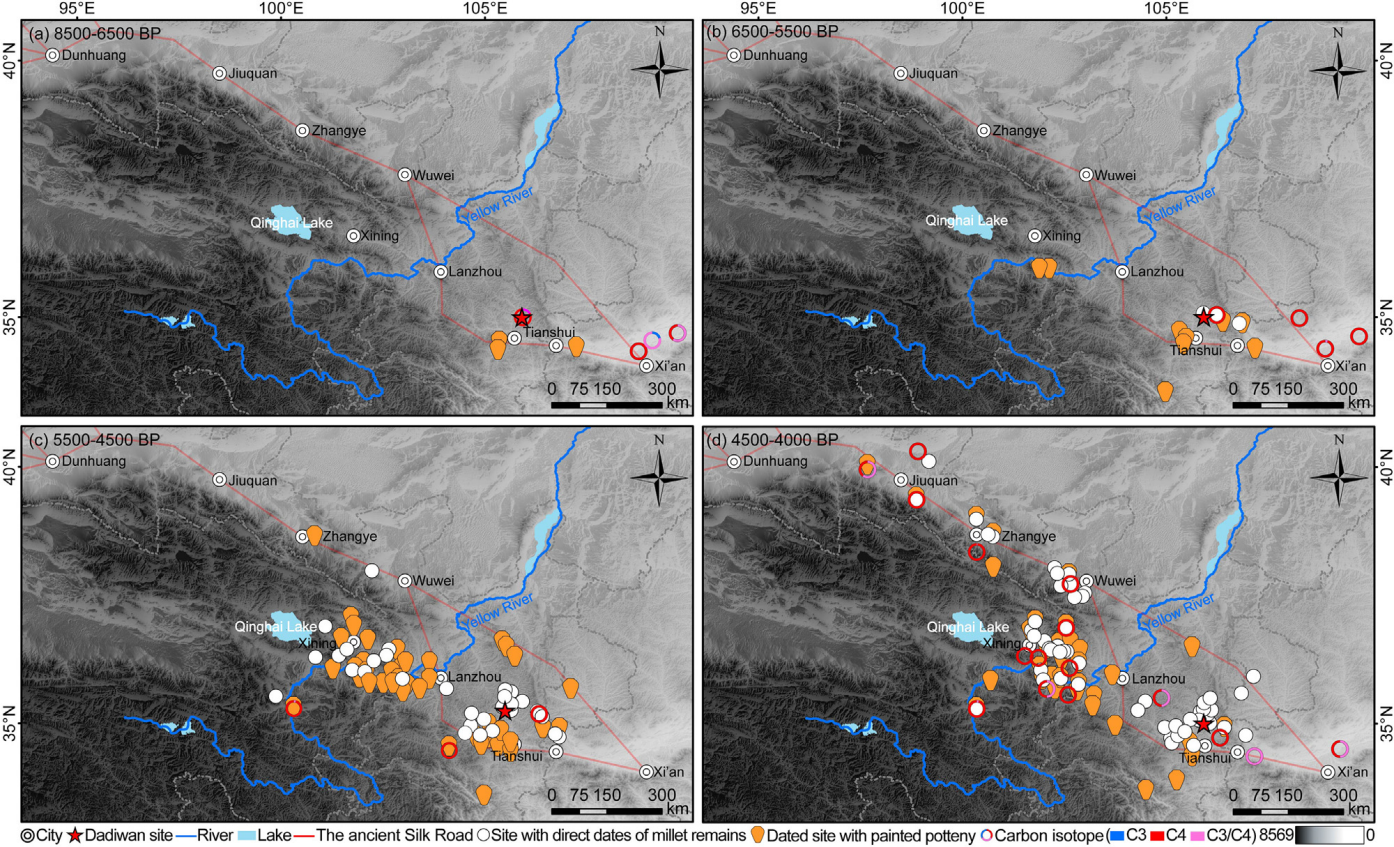
**The distribution of sites with painted pottery, direct millet dates, and stable carbon isotope data, between 8000 and 4000 BP along the ESR**.

Millet remains and painted pottery first appear at Dadiwan and the surrounding areas on the west side of Liupan Mountain between 7900 and 7200 BP. This studies archeobotanical analysis found 93 millet remains from the Paleolithic and the Dadiwan I cultural layers (L134-L33) (Table S1; Fig. 2). In addition, a single broomcorn millet grain from a microlithic layer (L48) was to 7922–7691 cal BP (Table 1; Fig. 5a). Previous studies reported eight broomcorn millet grains from the Dadiwan I cultural layers (7900–7200 cal BP), although these remains were not directly dated [62]. Painted pottery is also found at Dadiwan and the surrounding core site during this phase (7900–7200 cal BP), including the Laoguantai, Beishouling, Shizhaocun, and Xishanping sites (Figs. 1, 5a). Despite the presence of these cultivars in these core sites of early agricultural origins, during this period, the subsistence strategies of the ancestors at Dadiwan and its surrounding core sites remained predominantly gathering and hunting, with only the limited cultivation of plants such as millet. This is supported by the stable carbon isotope analysis of human bone collagen from the Yuhuazhai, Baijia and Beiliu sites, which found that the contribution of C4 plants such as millet to human diets was relatively limited [87,98,99]. Therefore, although millet utilization was limited during the pre-Yangshao period (7900–7200 cal BP), this initial appearance likely represents the first wave of dry-land agriculture expansion along the ESR, extending to the Dadiwan site and its surrounding areas (Figs. 1, 5a).

Painted pottery and millets have been found at numerous sites in the Tianshui area of Gansu Province dating to the early-mid Yangshao period (6500–5500 BP). These include the Dadiwan (this study), Shizhaocun [100], Xishanping [101,102], Gedachuan [52,91] and Huidier sites [94]. Direct radiocarbon dates of millets during the early Yangshao period are rare, limited to one millet sample from Dadiwan (6403–6297 cal BP) and one millet sample from Gedachuan (5997–5990 cal BP); however, sites containing painted pottery dating to the same time period are more common. This includes dated ceramics from Shizhaocun (6300–5929 cal BP), Xishanping (6395–6204 cal BP), and Huidier (6177–5327 cal BP) (Fig. 5b; Tables 1, S2). Simultaneously, both this analysis and a study by Liu [62] have unearthed substantial numbers of millet remains from Early Yangshao cultural layers at Dadiwan, suggesting that dry-land farming underwent initial intensification and likely expansion during this time. This interpretation is supported by stable carbon isotope analysis of human and animal bones at the Gedachuan and Dadiwan sites, which found an increase in C4 millet consumption after 6300 BP [52,67]. This wave of dry-land millet cultivation may have expanded to the edge of the northeastern Tibetan Plateau. Excavations from the Shalongka site found grain evidence (millet) and early-mid Yangshao painted pottery (6500–5500 cal BP) [103]. Painted pottery has also been discovered at the Andaqiha site on the edge of the northeastern Tibetan Plateau, with stratigraphic layers ranging from 6175 to 5605 cal BP (Table S2; Figs. 1, 5b). From this evidence, we argue that a second wave of dry-land agriculture likely expanded westward along the ESR during the early-mid Yangshao period between 6500 and 5500 cal BP. These people reached the area around modern-day Tianshui in Gansu Province, potentially extending to the eastern edge of the northeastern Tibetan Plateau (Figs. 1, 5b). This aligns with scholars who have proposed that the westward expansion of dry-land agriculture after 6500 cal BP is directly related to the expansion of the Yangshao culture [10,[104], [105]].

During the late Yangshao to early Majiayao periods (5500–4800 BP), painted pottery and dated millet are found in abundance along the ESR, particularly in the eastern edge of the Tibetan Plateau [106]. Direct dating of millet from multiple sites in the eastern margin of the Tibetan Plateau, their occupation and millet cultivation during the late Yangshao to early Majiayao periods. These sites include Hurere (5440–4973 cal BP), Gayixiangjing (5276–4861 cal BP), Luowalinchang (5285–4978 cal BP), Hongtujiaozi (5210–4861 cal BP), Baopo (5294–5045 cal BP), Yangwa (5283–4962 cal BP), Andaqiha (5038–4837 cal BP), Shalongka (5036–4838 cal BP) and Zhangga (5036–4838) (Figs. 1, 5c; Table S2). In addition, numerous sites with painted pottery sherds from this time period have been found in the same region. These sites include Hulijia (5583–5316 cal BP), Andaqiha (5300–4975 and 5281–4975 cal BP), Xinjia (5578–5311 and 5280–4843 cal BP), Lijiatai (5597–4978 and 5316–4975 cal BP), Shangsunjiazhai (5285–4620 cal BP), Lagongma (5285–4870 cal BP), Shangduoba (5304–4703 cal BP), Luowalinchang (5452–4989 cal BP) and Changning (5285–4864 cal BP) (Figs. 1, 5c; Table S2). Moreover, dietary evidence from stable carbon isotope analysis found notable C4 signals at the Zongri and Shannashuzha sites (5400–4800 BP), indicating that dry-land agriculture has expanded westward into the northeastern Tibetan Plateau (Figs. 1, 5c) [107,108].

This expansion of dry-land agriculture is found west along the ESR to the eastern and central Hexi Corridor (Fig. 1). Foxtail and broomcorn millet remains are found at the Gaomuxudi site in the Jiuquan area of the central Hexi Corridor, with the direct date from foxtail millet of 4825–4576 cal BP [109]. Painted pottery was found at the Donghuishan site in Zhangye area in the eastern part of Hexi Corridor in Gansu Province, dating between 5590 and 4625 cal BP [110]. Scholars argue that the westward spread of this dry-land agricultural system was driven by the expansion of the late Yangshao-early Majiayao cultures [107,[110], [111], [112]]. This expansion is linked with the intensification of high-yielding foxtail millet agriculture. This intensification, in turn, led to an increase in the number and size of late Yangshao-early Majiayao sites, propelling the large-scale westward expansion of dry-land agriculture. Our analysis suggests a third wave of dryland agricultural expansion westward along the ESR occurring during the late Yangshao to early Majiayao period (5500 to 4800 BP). This wave reached the eastern edge of the northeastern Tibetan Plateau and the central part of the Hexi Corridor (Figs. 1, 5c).

During the late Majiayao-Qijia period (4500–4000 BP), sites with painted pottery and millet are found across the Hexi Corridor (Figs. 1, 5d). Millet has been found at numerous late Majiayao-Qijia sites (4500–4000 BP), many with direct radiocarbon dates of grains. This includes the Xihetan (4403–3899 cal BP), Xichengyi (4227–3987 cal BP), Guojiashan (4413–4243 cal BP), Mozuizi (4235–3985 cal BP), Xitai (4146–3932 cal BP), Shuikou (4141–3934 cal BP), Duojialiang (4150–3985 cal BP) and Qipanshan sites (4146–3983 cal BP) (Figs. 1, 5d; Table S2). In addition, dietary evidence from the stable carbon isotope analysis of human remains from multiple late Majiayao-Qijia period cultural sites, including Wuba (4074–3486 cal BP), Huoshiliang (4087–3720 cal BP), Xichengyi (4227–3987 cal BP) and Mozuizi (4235–3985 cal BP), indicates the increased consumption of C4 plants, confirming high millet consumption (Figs. 1, 4d; Table S2). Characteristic painted ceramics are also found at multiple late Majiayao-Qijia sites in the Hexi Corridor, including the Taerwan, Yuanyangchi, Sibatan, Zhaobitan, Huoshaogou, Xitugou, and Xihetan sites (Figs. 1, 4d; Table S2). Notably, this wave of dry-land agriculture expanded along the ancient Silk Road as far as the Hami and Lop Nur regions of Xinjiang and eastern Central Asia. In southern Xinjiang, the remains of broomcorn millet have been unearthed at the Xiaohe Cemetery and Xintala site, with broomcorn millet in Xiaohe Cemetery directly dated to 3960–3704 cal BP [113,114]. In western Xinjiang, broomcorn millet directly dating to 4410–4225 cal BP was recovered from the Tongtiandong site [57], and in Eastern Kazakhstan, broomcorn millet dates to 4410–4103 cal BP at the Begash site [115]. This evidence indicates that dry-land millet agriculture reached eastern Central Asia by 4400 cal BP, and in the Hami and Lop Nur regions in southern Xinjiang by 3800 cal BP [12]. Some scholars have proposed that this expansion of dry-land agriculture may be directly related to the westward expansion of late Majiayao and Qijia culture from the Gansu-Qinghai area [78,[95], [96], [97]]. With the appearance of greater numbers of late Majiayao-Qijia sites (4500–4000 BP), we suggest this fourth wave of dry-land agriculture expansion occurred during the late Majiayao-Qijia period (4500–4000 BP), where it intersected with wheat and barley-based agriculture by 4400 cal BP, ultimately reaching the westernmost end of the ESR to northwestern Xinjiang and eastern Central Asia (Figs. 1, 5d).

The analysis indicates that the spread of dry-land millet agriculture on the ESR is linked with the successive expansions of the pre-Yangshao, early-mid Yangshao, late Yangshao-early Majiayao, and late Majiayao-Qijia cultures. With the increasing number of sites and population size, these linked practices were transmitted along the ancient Silk Road. Some scholars argue that these people supported the expansion of the Sino-Tibetan language family [43,116,117]. Investigating this linguistic movement, Zhang et al. [118], using Bayesian computational methods, calculated that Chinese and Tibetan speaking groups first diverged around 8000 BP. The “northern origin” hypothesis for the Sino-Tibetan language family argues that ancient populations in the upper reaches of the Yellow River during the early Yangshao culture migrated westward and eastward beginning by c. 6000 BP, and are the common ancestors of Sino-Tibetan language populations [119,120]. These linguistic studies coincide with the large-scale utilization of millet found at Dadiwan. These analysis findings are consistent with the “farming/language dispersal” hypothesis, which argues that agricultural development is an important driving force for the origin and diffusion of languages. Several scholars utilizing a phylogenetic analysis of Sino-Tibetan languages also argue for their origin in the middle and upper reaches of the Yellow River during the Neolithic, an argument in line with some archeological evidence [121,122]. In contrast to the “farming/language” model of the spread of Sino-Tibetan languages, some researchers argue that the expansion of Indo- European language populations on the Eurasian grasslands relied on horses and carriages for rapid migration and dissemination [[123], [124], [125], [126]], in addition to research variously arguing for factors such as climate, trade development, and disease outbreaks leading to migration and language transmission [[127], [128], [129]].

## 6 Conclusion

The earliest evidence for millet utilization in the ESR region dates to approximately 7900 cal BP, based on systematic archeobotanical studies and the direct radiocarbon dating of 29 millet grains from the Dadiwan site. Our findings reveal that, between ∼7900 and 7200 cal BP, millets were initially cultivated only occasionally, with increasing use between ∼6500 and 5500 cal BP, after which foxtail millet cultivation became a dominant component of subsistence between ∼5500 and 4800 BP. In addition, 10 of the 29 dated millet samples at Dadiwan are inconsistent with the relative chronologies of their associated cultural strata, potentially due to a combination of taphonomic factors. This result foregrounds the necessity to directly date carbonized grains to reliably reconstruct the chronologies of prehistoric agriculture systems.

This study integrates new direct radiocarbon dates of charred millet with previously published radiocarbon dates and dietary evidence from stable carbon isotope analysis indicating C4 plant consumption. When applied to existing archeological datasets from Neolithic sites across the ESR, this data leads us to argue for four waves of expansion for dry-land millet cultivation. Dry-land agriculture reached the eastern end of the ESR during the pre-Yangshao period (7900–7200 BP), extending west to Dadiwan around 7900 BP, where it supplemented existing subsistence strategies. Between ∼6500 and 5500 BP, dry-land millet agriculture became a primary subsistence strategy in the Wei River valleys of the eastern ESR, forming a component of early-mid Yangshao culture, and potentially extending to the edge of the northeastern Tibetan Plateau. This millet-based agricultural system expanded west to the eastern margin of the Tibetan Plateau and central Hexi Corridor between ∼5500 and 4500 BP, simultaneous with the expansion of the late Yangshao and early Majiayao cultures. Between ∼4500 and 4000 BP, rainfed millet agriculture extended into the western ESR, an expansion associated with the Majiayao and Qijia cultures.

## CRediT authorship contribution statement

**Haiming Li:** Writing – review & editing, Writing – original draft, Methodology, Investigation, Funding acquisition, Formal analysis, Conceptualization. **Yingyu Qian:** Software, Investigation. **Zeli Wang:** Visualization, Software, Data curation. **Nathaniel James:** Writing – review & editing, Formal analysis, Data curation. **Yifu Cui:** Methodology, Investigation, Data curation. **Yishi Yang:** Methodology, Investigation, Formal analysis. **Xin Jia:** Writing – review & editing, Writing – original draft, Validation, Formal analysis, Conceptualization. **Guanghui Dong:** Writing – review & editing, Writing – original draft, Resources, Project administration, Investigation, Formal analysis, Conceptualization.

## Declaration of competing interest

The authors declare that they have no conflicts of interest in this work.

## Appendix Supplementary materials

Image, image 1 dummy alt text

Image, application 2

Image, application 3
