# Supplementary figures and images for: Multiple waves of westward dry-land agriculture expansions along the East Silk Road during the Neolithic age
Source: Fundam Res. 2026 Jan 12;6(3):1321–31. doi: 10.1016/j.fmre.2025.12.013 (PMC13247478; doi:10.1016/j.fmre.2025.12.013)

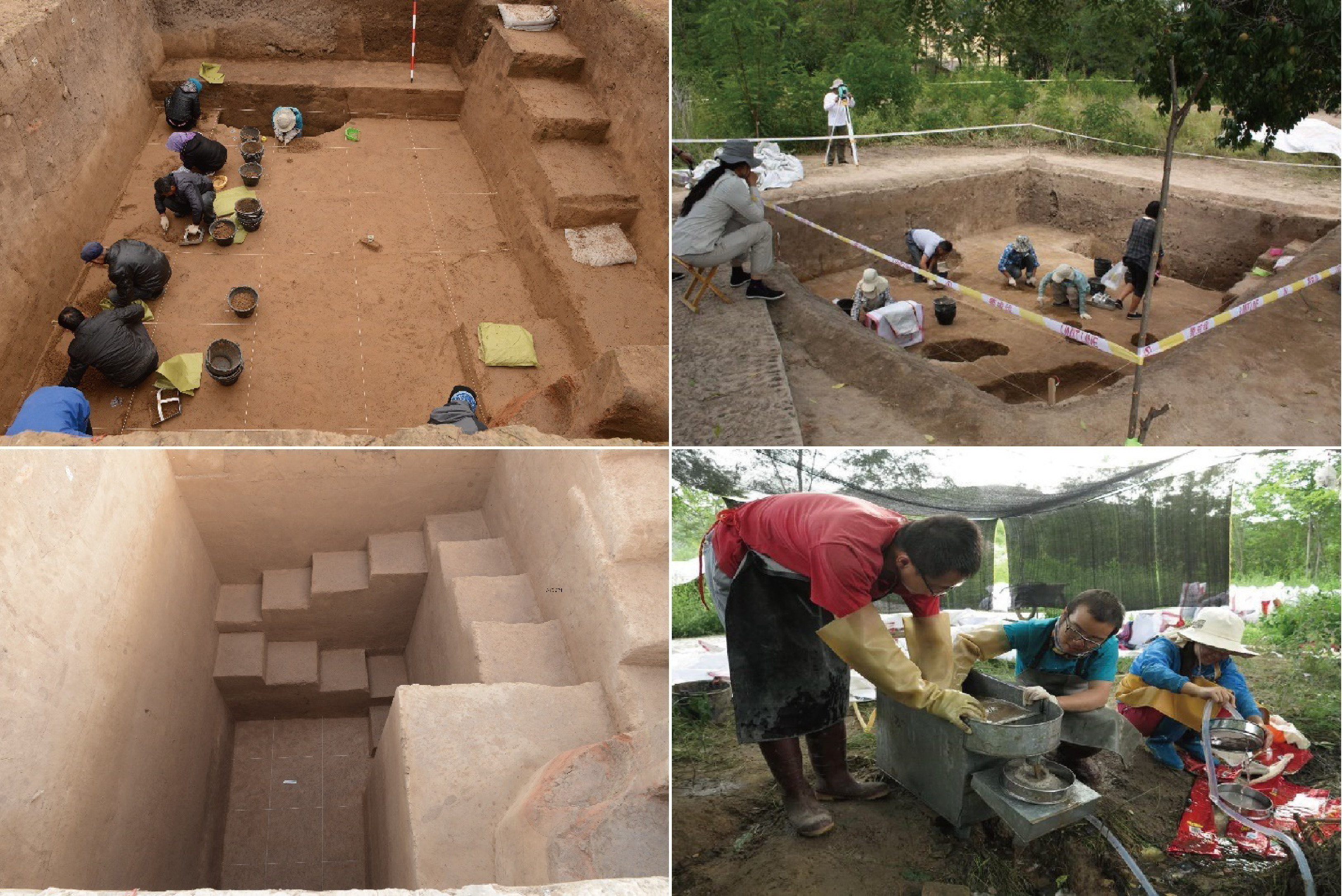

Supplement: Supplementary file 1 [file mmc1.jpg]
