# Supplementary material for: Multiple waves of westward dry-land agriculture expansions along the East Silk Road during the Neolithic age
Source: Fundam Res. 2026 Jan 12;6(3):1321–31. doi: 10.1016/j.fmre.2025.12.013 (PMC13247478; doi:10.1016/j.fmre.2025.12.013)
Supplement: Supplementary file 2 [file mmc2.docx]

**Supplementary Table 1 Assemble of identified plant remains from the excavation of the Dadiwan site.**

| **Sample Level/feature** | **Soil (L)** | **Agriculture crops** | | | **Weeds** | | | | | | | | | | | | | | **Total** |
| --- | --- | --- | --- | --- | --- | --- | --- | --- | --- | --- | --- | --- | --- | --- | --- | --- | --- | --- | --- |
|  |  | ***Setaria italica*** | ***Panicum miliaceum*** | ***Triticum aestivum*** | ***Glycine max*** | ***Setaria viridis*** | ***Poa annua L.*** | ***Astragalus adsurgens*** | ***Melilotus suaveolens*** | ***Chenopodium album*** | ***Atriplex spp.*** | ***Kochia scoparia*** | ***Polygonum nepalense*** | ***Rumex acetosa*** | [***Vicia cracca***](javascript:;) | ***Glycyrrhiza uralensis*** | ***Hippophae rhamnoides*** | ***Galium tricorne*** |  |
| L12 | 85 | 56 | 37 |  |  |  |  |  |  | 4 |  |  |  |  |  |  | 1 | 1 | 99 |
| L13 | 144 | 625 | 97 |  |  |  |  |  |  |  |  |  |  |  |  |  |  | 1 | 723 |
| L14 | 248 | 864 | 227 |  |  |  |  |  | 1 | 16 |  |  |  | 1 |  | 1 |  | 1 | 1111 |
| L15 | 224.8 | 1010 | 242 |  |  |  |  |  |  | 3 |  |  |  | 1 |  |  |  |  | 1256 |
| L16 | 46 | 101 | 75 |  |  |  |  | 1 |  | 5 |  |  |  | 1 |  |  |  | 1 | 184 |
| L17 | 95 | 113 | 60 | 1 |  |  |  |  |  | 2 |  |  |  | 2 |  |  |  |  | 178 |
| L18 | 8 | 14 | 11 |  |  |  |  |  |  | 1 |  |  |  |  |  |  |  |  | 26 |
| L19 | 401.7 | 1397 | 229 | 1 | 1 |  |  |  |  | 13 |  |  |  |  |  |  |  |  | 1641 |
| L20 | 198.4 | 259 | 108 |  |  |  |  |  |  |  |  |  |  |  |  |  |  |  | 367 |
| L21 | 257.4 | 452 | 101 |  |  |  |  |  |  | 3 |  |  |  |  |  |  |  |  | 556 |
| L22 | 284.5 | 232 | 89 |  |  | 1 |  |  |  | 7 |  |  | 1 |  |  |  |  | 2 | 332 |
| L23 | 198 | 107 | 44 |  |  |  |  |  |  | 8 |  |  |  |  |  | 1 |  |  | 160 |
| L24 | 272.5 | 91 | 28 |  |  |  |  |  |  | 9 |  |  |  |  |  |  |  |  | 119 |
| L25 | 300 | 217 | 44 | 1 |  |  |  |  |  | 4 |  |  |  |  |  |  |  | 1 | 267 |
| L26 | 357.2 | 215 | 34 | 1 |  |  |  |  |  | 2 |  |  |  |  |  |  |  |  | 252 |
| L27 | 404 | 62 | 26 |  |  |  |  |  |  | 8 | 1 |  |  |  |  | 1 |  |  | 98 |
| L28 | 450.5 | 58 | 20 | 1 |  |  |  |  |  | 4 |  |  |  |  |  |  |  |  | 82 |
| L29 | 418.5 | 27 | 16 |  |  |  |  |  | 1 | 1 |  |  |  |  |  |  |  |  | 45 |
| L31 | 405 | 19 | 4 |  |  |  |  |  |  |  |  |  |  |  |  |  |  |  | 23 |
| L32 | 390.5 | 41 | 10 | 1 |  |  |  |  |  | 1 |  |  |  |  |  |  |  |  | 53 |
| L33 | 372.5 | 13 | 10 |  |  |  |  |  |  |  |  |  |  |  |  |  |  |  | 23 |
| L34 | 412 | 14 | 8 |  |  |  |  |  |  | 7 |  |  |  |  |  |  |  |  | 29 |
| L35 | 362.5 | 11 | 14 |  |  |  |  |  |  | 1 |  |  |  |  |  |  |  |  | 26 |
| L36 | 296 | 7 | 3 |  |  |  |  |  |  | 1 |  |  |  |  |  |  |  |  | 11 |
| L37 | 243 | 3 | 1 |  |  |  |  |  |  |  |  |  |  |  |  |  |  |  | 4 |
| L39 | 266 | 1 | 1 |  |  |  |  |  |  |  |  |  |  |  |  |  |  |  | 2 |
| L40 | 262 | 3 |  |  |  |  |  |  |  |  |  |  |  |  |  |  |  |  | 3 |
| L42 | 213 |  | 1 |  |  |  |  |  |  |  |  |  |  |  |  |  |  |  | 1 |
| L48 | 186 |  | 1 |  |  |  |  |  |  |  |  |  |  |  |  |  |  |  | 1 |
| L52 | 167 |  | 1 |  |  |  |  |  |  |  |  |  |  |  |  |  |  |  | 1 |
| L59 | 214 |  | 1 |  |  |  |  |  |  |  |  |  |  |  |  |  |  |  | 1 |
| F1 floor 0 | 41 | 4 | 28 |  |  |  |  |  |  | 6 |  |  | 1 |  |  |  |  |  | 39 |
| F1 floor 1 | 227.8 | 39 | 115 |  |  |  |  | 1 | 1 | 22 |  |  |  | 2 |  |  |  |  | 180 |
| F1 floor 2 | 212.8 | 55 | 122 |  |  |  |  |  |  | 18 |  | 1 | 1 |  |  |  |  |  | 197 |
| F1 floor 3 | 258 | 65 | 220 |  |  |  |  | 2 |  | 15 |  |  | 1 | 4 |  |  | 2 | 2 | 311 |
| F1 floor 4 | 433.8 | 137 | 917 | 1 | 1 |  |  |  | 1 | 30 |  |  | 4 | 7 |  | 3 | 1 | 9 | 1111 |
| F1 floor 5 | 94.5 | 13 | 14 |  |  |  |  |  |  | 11 |  | 1 |  |  |  |  |  | 2 | 41 |
| H6 | 1481.7 | 864 | 2043 |  | 1 |  |  |  |  | 127 |  |  | 9 | 12 |  | 48 | 2 | 14 | 3120 |
| H1 | 18 | 5 | 4 |  |  |  |  |  |  |  |  |  |  |  |  |  |  |  | 9 |
| F2 floor 1 | 32.8 | 22 | 20 |  |  |  |  |  |  | 14 |  |  |  |  |  |  |  |  | 56 |
| F2 floor 2 | 65 | 67 | 25 |  |  |  |  |  |  | 6 |  |  |  | 3 |  | 1 |  | 1 | 103 |
| F2 floor 3 | 78.5 | 61 | 25 |  |  |  |  |  |  | 4 |  |  |  | 1 |  |  |  |  | 91 |
| F2 floor 4 | 22 | 13 | 12 |  |  |  |  |  |  |  |  |  | 1 |  |  |  | 1 | 1 | 28 |
| F2 | 996.8 | 1387 | 1143 |  | 1 |  | 1 | 6 | 2 | 124 |  |  |  | 184 |  | 38 | 2 | 3 | 2891 |
| H2 | 153 | 2251 | 1198 |  |  | 1 |  | 3 | 2 | 27 | 1 | 1 |  | 2 | 1 | 2 |  | 6 | 3495 |
| H4 | 132.5 | 1352 | 182 |  |  | 1 |  |  | 3 | 1 |  |  |  | 3 |  |  |  |  | 1542 |
| F3 | 42.5 | 5 | 10 |  |  |  |  |  |  |  |  |  |  |  |  |  |  |  | 15 |
| FT9 | 297 | 792 | 161 |  |  |  |  | 2 |  | 1 |  |  |  | 1 |  |  |  |  | 957 |
| G1 | 1121 | 254 | 502 |  | 2 |  |  | 2 |  | 94 | 1 | 1 |  | 2 | 3 | 1 | 1 | 9 | 872 |
| H5 | 157 | 169 | 170 |  |  |  | 1 |  |  | 2 |  | 1 | 2 | 4 |  |  |  |  | 349 |
| H6 | 65.5 | 1140 | 785 |  | 2 | 1 |  |  |  | 17 | 1 |  | 3 |  |  | 1 |  | 1 | 1951 |
| H7 | 130 | 195 | 119 |  |  |  |  |  |  | 3 |  |  |  |  |  |  |  |  | 317 |
| F2-ZD1 | 120 | 15 | 20 |  |  |  |  |  |  | 1 |  |  |  |  |  |  |  | 2 | 38 |
| F2-ZD3 | 32 | 3 | 11 |  |  |  |  |  |  | 11 |  |  |  |  |  |  |  | 2 | 27 |
| Total | 14396.2 | 14921 | 9388 | 7 | 8 | 4 | 2 | 17 | 11 | 634 | 4 | 5 | 23 | 230 | 4 | 97 | 10 | 59 | 25424 |
